# Synthesis and Stabilization of Gold Nanoparticles Using Water-Soluble Synthetic and Natural Polymers

**DOI:** 10.3390/polym12112625

**Published:** 2020-11-08

**Authors:** Zhanara A. Nurakhmetova, Aiganym N. Azhkeyeva, Ivan A. Klassen, Gulnur S. Tatykhanova

**Affiliations:** 1 Institute of Polymer Materials and Technology, Almaty 050013, Kazakhstan; ayganym_90@mail.ru (A.N.A.); merkrunner123@gmail.com (I.A.K.); gulnur-ts81@yandex.kz (G.S.T.); 2 Laboratory of Engineering Profile, Satbayev University, Almaty 050013, Kazakhstan

**Keywords:** gold nanoparticles (AuNPs), gold nanorods (AuNRs), one-pot and growth seeding methods, polysaccharides, synthetic water-soluble polymers, storage stability

## Abstract

Gold nanoparticles (AuNPs) were synthesized and stabilized using the one-pot method and growth seeding, through utilization of synthetic polymers, including poly(*N*-vinylpyrrolidone) (PVP), poly(ethylene glycol) (PEG), and poly(vinylcaprolactame) (PVCL), as well as natural polysaccharides, including gellan, welan, pectin, and κ-carrageenan. The absorption spectra, average hydrodynamic size, ζ-potential, and morphology of the gold nanoparticles were evaluated based on various factors, such as polymer concentration, molecular mass of polymers, temperature, and storage time. The optimal polymer concentration for stabilization of AuNPs was found to be 4.0 wt % for PVP, 0.5 wt % for gellan, and 0.2 wt % for pectin, welan, and κ-carrageenan. The values of the ζ-potential of polymer-stabilized AuNPs show that their surfaces are negatively charged. Most of the AuNPs are polydisperse particles, though very monodisperse AuNPs were detected in the presence of a 0.5 wt % gellan solution. At a constant polymer concentration of PVP (4 wt %), the average size of the PVP–AuNPs decreased with the decrease of molecular weight, and in the following order: PVP 350 kDa (~25 nm) > PVP 40 kDa (~8 nm) > PVP 10 kDa (~4 nm). The combination of Fourier-transform infrared spectroscopy (FTIR) and Raman spectroscopy revealed that the functional groups of polymers that are responsible for stabilization of AuNPs are lactam ring in PVP, carboxylic groups in gellan and welan, esterified carboxylic groups in pectin, and SO_2_ groups in κ-carrageenan. Viscometric and proton nuclear magnetic resonance (^1^H NMR) spectroscopic measurements showed that the temperature-dependent change in the size of AuNPs, and the gradual increase of the intensity of AuNPs at 550 nm in the presence of gellan, is due to the rigid and disordered conformation of gellan that affects the stabilization of AuNPs. The AuNPs synthesized in the presence of water-soluble polymers were stable over a period of 36 days. Preliminary results on the synthesis and characterization of gold nanorods stabilized by polymers are also presented.

## 1. Introduction

Hydrophilic water-soluble polymers of synthetic and natural origin have attracted increasing interest as stabilizers for AuNPs because they exhibit many advantages, such as enhanced long-term stability, compatibility, and processability [1,2,3]. The low inherent toxicity, multifunctionality, high surface area, and photophysical and optical properties of AuNPs impart unique attributes that have a great importance in catalysis, chemotherapy, cancer diagnosis, and drug delivery [4,5]. The fabrication of AuNPs is extremely important for various nanotechnology-related biomedical applications, considering their non-toxicity, stability, chemical inertness, and unique optical, physicochemical, and biological properties [6,7]. Thus, colloidal gold is applicable in various medical-related research fields, including biosensing and bio-detection, bioelectronics, and selective tumor imaging and targeting [8,9,10,11].

A wide array of solution-based approaches has been developed in the past few decades to control the size, shape, and surface functionality of AuNPs [12]. AuNPs can undergo irreversible aggregation during storage time. Some strategies have been developed to solve this problem using surfactants, polymers, and proteins, among others. Polymer-protected AuNPs possess higher stability compared to most conventional AuNP fabrication methods, due to the electrostatic stabilization based on the double layer repulsion between the particles, and the steric stabilization of colloidal particles achieved by physical adsorption of macromolecules on the surface of the particles [13]. AuNPs are commonly engineered with polymeric coatings to improve their physicochemical properties. The use of natural biodegradable polymers for stabilization of AuNPs has potential in the field of nanomedicine, in order to improve biocompatibility, bioavailability, and safety, due to the low toxicity of AuNPs [14,15].

Among the variety of different shapes of AuNP: spheres, shells, rods, stars, tubes, wires, and so forth, the most suitable form for application in plasmonic photothermal therapy (PPTT) is the gold nanorod (AuNR) [16,17]. PPTT is currently one of the most promising research avenues in the treatment of cancer cells and infectious diseases. The essence of PPTT is that AuNRs have absorption maxima in the visible or near-IR (NIR) region, and become hot when irradiated with light. If AuNRs are located inside or around the targeted cells, which can be achieved by conjugating gold nanoparticles to antibodies or other molecules, these cells can be eliminated. Thermal cancer therapy is done with 20–40 nm AuNRs, which convert 20 nanosecond laser irradiation (λ = 514 nm) to local heat (up to 40–45 °C), resulting in the selective death of cancer cells. PPTT [18] has been extensively researched and used for biomedical applications [19]. In a recent review [20], advancements in plasmonic nanoparticles and films in the field of biomedicine were examined.

AuNPs can be synthesized and stabilized by many proteins, such as bovine serum albumin, lysozyme, and keratin, among others [21,22,23,24,25,26]. The conjugation of AuNPs with biomolecules is relevant in medicinal applications, such as imaging, sensing, and photothermal therapy, due to good biocompatibility, biodegradability, and low toxicity. Recently, gold nanoparticles were utilized for the detection of oxidation stress biomarkers [27], DNA carriers [28], and proteins, the latter of which was done with extreme accuracy using gated resonance energy transfer (gRET) [29]. However, in spite of achievements in the preparation, characterization, and application aspects of AuNPs stabilized by numerous synthetic and natural polymers, a completely “green” polysaccharide-based AuNP synthetic method is, to the best of our knowledge, still lacking [5,30].

The current paper is devoted to the synthesis, stabilization, and characterization of AuNPs and AuNRs using synthetic polymers, including poly(ethyeleneglycol) (PEG), poly(*N*-vinylpyrrolidone) (PVP), and poly(vinylcaprolactame) (PVCL), as well as certain natural polysaccharides, including gellan, welan, pectin, and κ-carrageenan.

## 2. Materials and Methods

Absorption spectra of AuNPs and AuNRs were determined at various temperatures using UV–Vis spectroscopy (Specord 210 plus BU, Jena, Germany). The average hydrodynamic size and ζ-potential of gold nanoparticles were determined with the help of a dynamic light scattering (DLS) device (Malvern Zetasizer Nano ZS90, Malvern, UK) at pH ≈12, and without adjusted ionic strength. Shape and size of the AuNPs and AuNRs were determined using TEM micrographs (JEOL Ltd., JEM-1400 Plus, Tokyo, Japan). FTIR and Raman spectra were registered on Carry 660 (Agilent, Santa Clara, CA, USA) and NTEGRA Spectra spectrometers (NT-MDT, Zelenograd, Russia), using 473 nm excitation. ^1^H nuclear magnetic resonance (NMR) spectra of 1 wt % gellan solution were recorded, using JNM-ECA 400 (JEOL Ltd., Tokyo, Japan) in D_2_O at 30, 45 and 65 °C. A microwave reactor (Monowave 50 Anton Paar, Graz, Austria), equipped with a thermal and time controller, was used for synthesis of the AuNPs. The AuNR dispersions were separated from the supernatant using a centrifuge (Eppendorf 5810R, Tuttlingen, Germany).

**Figure 1:**
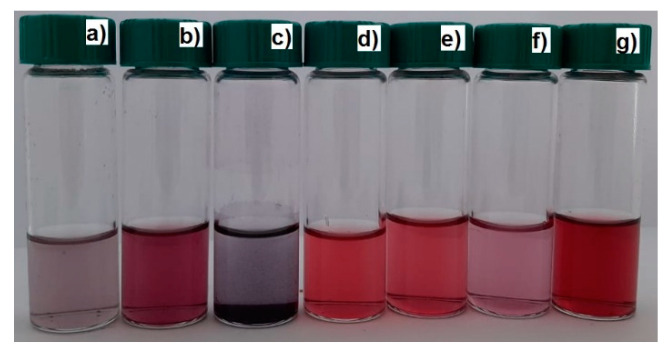
Photos of gold nanoparticle (AuNP) samples synthesized and stabilized using κ-carrageenan (**a**), PEG 40 kDa (**b**), gellan (**c**), PVP 10 kDa (**d**), PVP 40 kDa (**e**), pectin (**f**) welan (**g**) at 100 °C; [HAuCl_4_] = 0.01 mM.

AuNPs synthesized and stabilized using PVP, PEG, gellan, welan, pectin, and κ-carrageenan were obtained using the one-pot synthesis method [31]. For this 0.05, 0.2, 1, and 4 wt % polymer solutions, 0.33 mL of HAuCl_4_ (C = 100 mg·mL^−1^) and 4 mL of 0.5 M KOH were mixed, stirred, and heated up to 100 °C for 3–5 min in a microwave reactor (Monowave 50 Anton Paar, Austria). As a result, colored solutions, from yellow to wine red or purple, were formed due to formation of AuNPs (Figure 1). All the experiments were performed in triplicate, and the results were taken as average means ± standard deviations.

For preparation of polymer stabilized AuNPs for the FTIR and Raman measurements, the same experiments were carried out in the presence of 5 mL HAuCl_4_ (C = 100 mg·mL^−1^).

**Figure 2:**
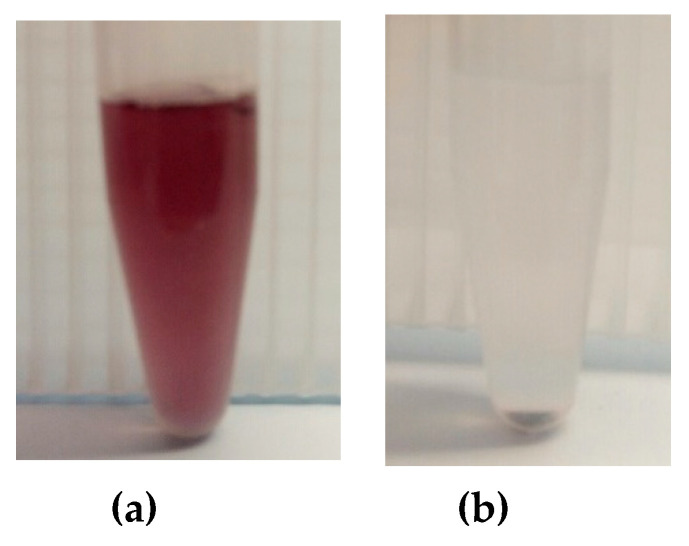
Separation of gold nanorods (AuNRs) dispersion from CTAB and by-products: (**a**) before centrifugation; (**b**) after centrifugation.

In order to obtain AuNRs, the seed-mediated growth method described in [32] was used. First, 5 mL of 0.2 M cetyl trimethylammonium bromide (CTAB) solution was mixed with 5 mL of 0.5 mM HAuCl_4_ and stirred. Then, 0.6 mL of freezing 0.01 M NaBH_4_ was added, which resulted in formation of a brownish-yellow solution of AuNPs. In the next stage, a mixture consisting of CTAB (30 mL, 0.2 M), AgNO_3_ (1.5 mL, 4 mM), and HAuCl_4_ (30 mL, 1 mM) was gently mixed and 0.42 mL of 78.8 M ascorbic acid was added. Ascorbic acid worked as a mild reducing agent, changing the growth solution from dark yellow to colorless. Finally, the first seed solution (72 µL) was added to the second growth solution, and the mixture was incubated at 30 °C overnight. As a result, a crimson solution, containing AuNRs, was obtained (Figure 2). In order to remove the by-products and CTAB from the AuNRs, the solution was centrifuged at 10,650 rpm for 30 min. Then, the precipitate was resuspended through the addition of 1 mL of deionized water, and centrifuged at 10,650 rpm for 15 min. After being washed twice, the AuNRs were resuspended and stabilized in 5 mL of selected polymer solutions.

## 3. Results and Discussion

### 3.1. Influence of the Nature of Polymers on the Average Hydrodynamic Size of AuNPs

The optimal conditions for synthesis of AuNPs were established by varying the nature, molecular mass, and concentration of the polymers. The synthetic polymers, including PVP with *M*_n_ = 10, 40, 350 kDa, and PEG with *M*_n_ = 6 and 40 kDa, and the polysaccharides, including gellan, welan, pectin, and κ-carrageenan, were used as polymeric stabilizing agents.

**Figure 3:**
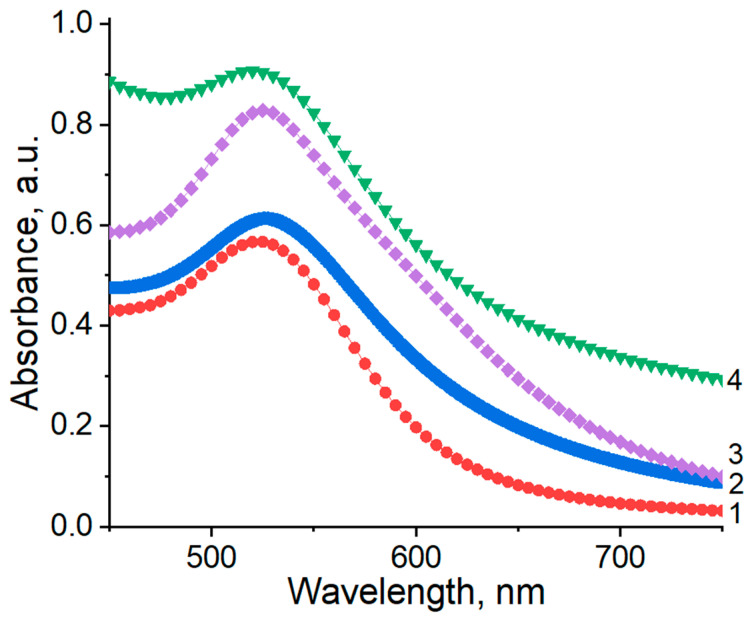
UV–Vis spectra of AuNPs synthesized and stabilized at 25 °C using synthetic and natural polymers. 1: PVP 40 kDa, 2: gellan, 3: κ-carrageenan, 4: pectin; [HAuCl_4_] = 0.01 mM.

UV–Vis spectroscopy is an effective technique for verifying the formation and stabilization of aqueous AuNPs. Usually, AuNPs exhibit an absorption band in the visible spectral region, which is known as surface plasmon resonance (SPR). Spherical AuNPs exhibit a range of colors (e.g., orange, red, and purple) in aqueous solution as the core size increases from 1 to 100 nm, and generally show a size-relative absorption peak from 500 to 550 nm [33,34,35]. The SPR is influenced, not only by size, but also by the shape, nature of the solvent, core charge, and temperature [36,37]. Aggregation of nanoparticles results in significant red-shifting of the SPR frequency, broadening of the surface plasmon band, and changing the solution color from red to blue because of interparticle plasmon coupling [38]. For example, the AuNPs without any encapsulating agent show an SPR band at 543 nm. In the presence of stabilizing polymers, the blue shift in the typical SPR band, which is centered at approximately 525 ± 2 nm, is ascribed to the quantum confinement effect, as shown in Figure 3 [36,39].

Size control of the AuNPs is an important aspect in colloidal gold synthesis. Various research studies have shown that the interaction of gold nanoparticles with polymers strongly impacts the size, stability, and distribution of nanoparticles [40]. Better biocompatibility, cytotoxicity, and catalytic properties may be expected when monodisperse polymer-coated AuNPs with smaller sizes are produced in a water medium. The average hydrodynamic size distributions and ζ-potentials of the AuNPs synthesized at optimally selected concentrations of synthetic and natural polymers are summarized in Table 1.

Based on distribution, most of the AuNPs were polydisperse. The average diameter of AuNPs in the presence of 4 wt % PVP solution ranged from 4–9 nm, a wider distribution of AuNPs was observed in the presence of pectin and κ-carrageenan. Monodisperse AuNPs with a size of 16 nm were formed in the presence of a 0.5 wt % gellan solution. Cancer cell growth is known to be faster in comparison to that of healthy cells, the faster metabolism of which consequently increases the glucose uptake rate [41]. The authors consider that gellan-AuNPs might be the most suitable for PPTT, because gellan gum, consisting of a tetrasaccharide repeating unit, could serve as a “reservoir” of sugar for the malignant cells during their accelerated growth and division. Therefore, the use of polysaccharide-coated particles could preferentially enhance the delivery of active agents into tumor cells. Conjugated with gellan, AuNPs that accumulate near tumor cells could be damaged and/or destroyed in the course of PPTT treatment.

**Figure 4:**
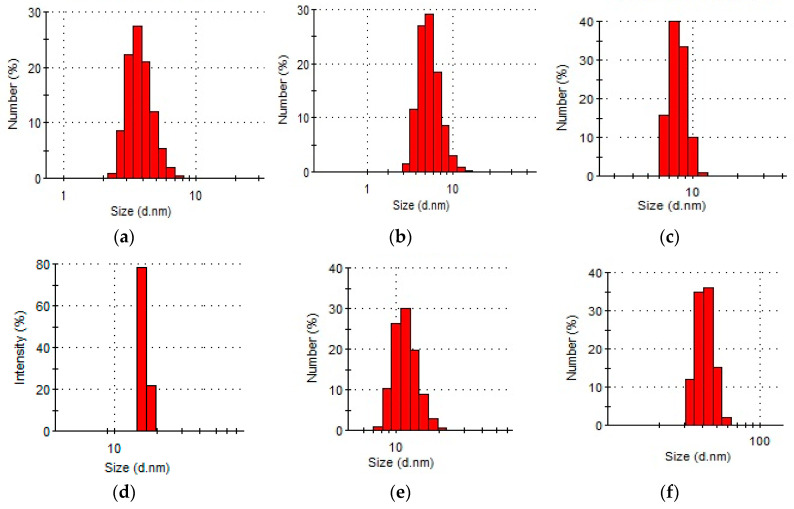
Average hydrodynamic size distribution of AuNPs stabilized by synthetic and natural polymers at 25 °C: (**a**) PVP 10 kDa, (**b**) PVP 40 kDa, (**c**) PEG 40 kDa (**d**) gellan, (**e**) κ-carrageenan, (**f**) welan; [HAuCl_4_] = 0.01 mM.

The DLS data of AuNPs synthesized and stabilized using various polymers, with optimal concentrations, are presented in Figure 4.

The surface charge of AuNPs plays a crucial role in the course of fabrication of nanosystems with potential applications in biomedicine [33]. The surface charge of AuNPs, estimated in terms of ζ-potential, facilitates their physicochemical stability and further implementation in cellular processes. Previous research studies have revealed that the toxicity level assigned to AuNPs is strongly dependent on the particle surface charge, thus positively charged AuNPs cause cell death at a lower concentration, while neutrally charged particles result in cell death at significantly higher concentrations. The values of the ζ-potential of polymer-stabilized AuNPs show that their surface is negatively charged (Table 1).

### 3.2. Influence of the Concentration and Molecular Mass of PVP on the Size of AuNPs

Selection of appropriate polymer concentration and molecular mass is very important for stabilization of AuNPs. We have found that the optimal concentration of PVP for stabilization of AuNPs is 4 wt % (Table 2).

**Figure 5:**
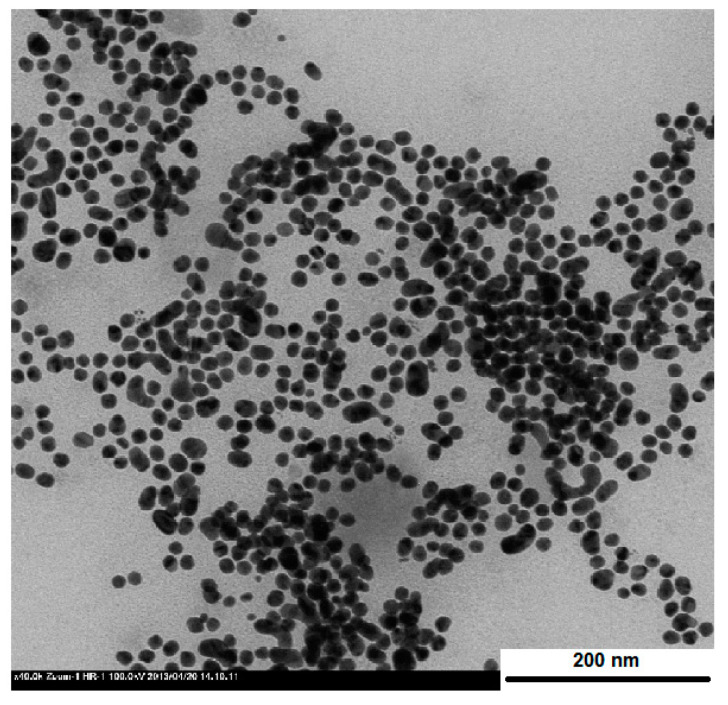
TEM image of AuNPs protected using PVP 40 kDa.

At a constant polymer concentration of PVP (4 wt %), the average size of PVP–AuNPs, depending on the molecular mass of the PVP variant, decreases in the following order: PVP 350 kDa (~25 nm) > PVP 40 kDa (~8 nm) > PVP 10 kDa (~4 nm). As revealed in the TEM image (Figure 5), the shape of AuNPs stabilized by PVP 40 kDa was spherical, with an average size 7 ± 1 nm, which is in good agreement with the results of DLS (d = 8 ± 1 nm).

### 3.3. FTIR and Raman Spectra of AuNPs Stabilized Using Synthetic and Natural Polymers

**Figure 6:**
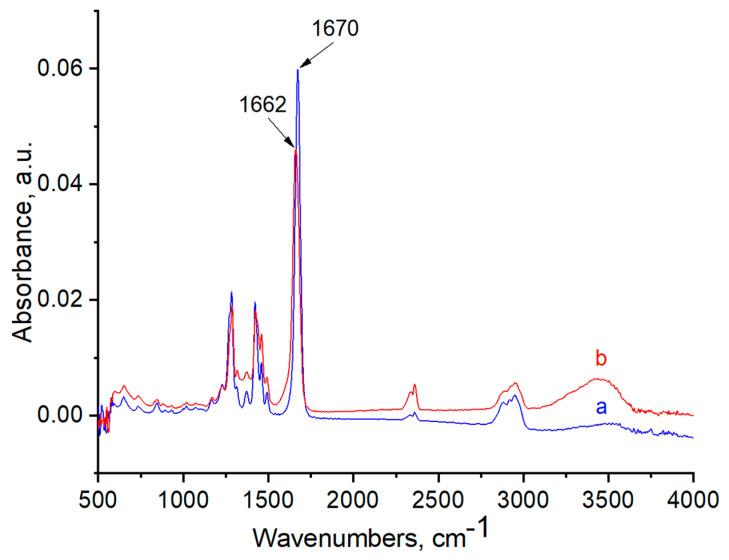
FTIR spectra of PVP 40 kDa (**a**) and PVP-AuNPs (**b**).

**Figure 7:**
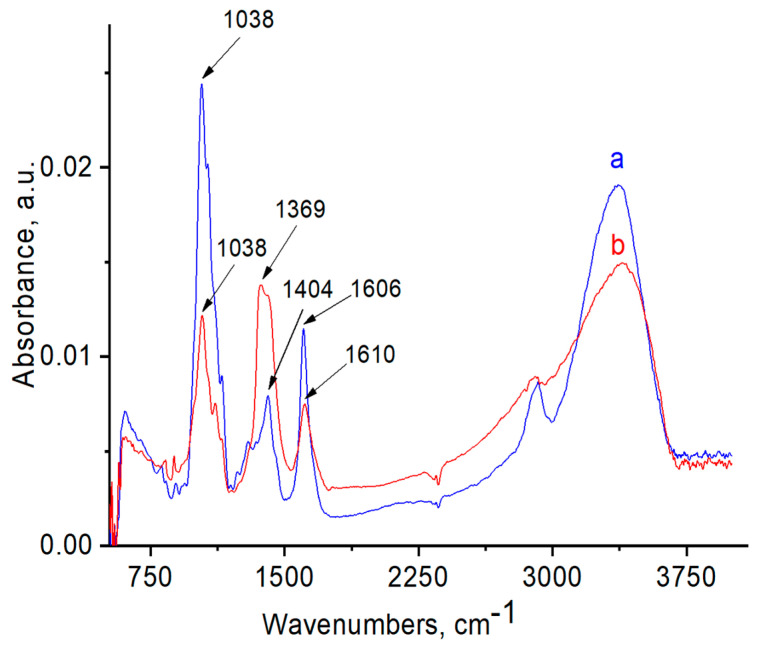
FTIR spectra of gellan (**a**) and gellan-AuNPs (**b**).

**Figure 8:**
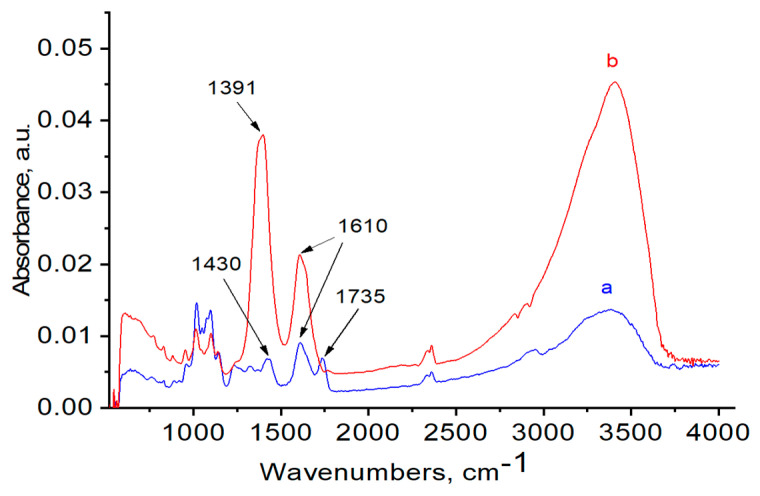
FTIR spectra of pectin (**a**) and pectin-AuNPs (**b**).

**Figure 9:**
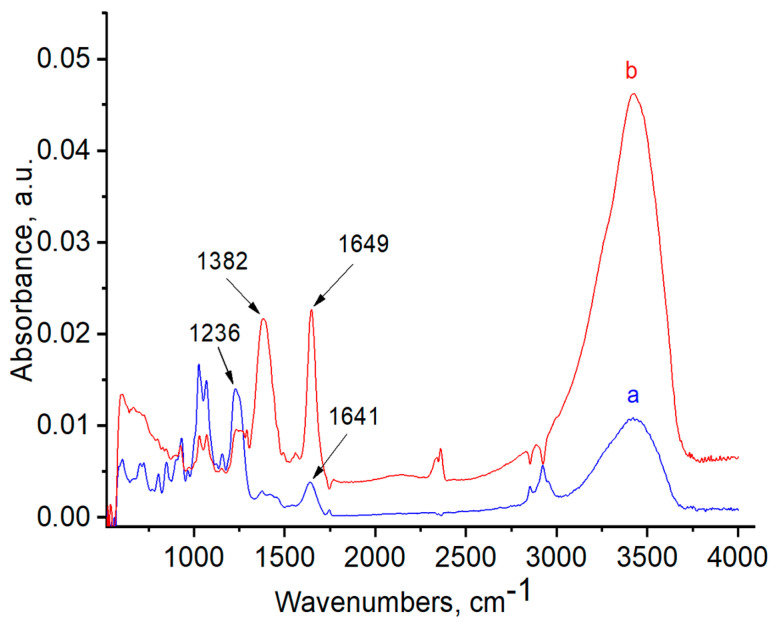
FTIR spectra of κ-carrageenan (**a**) and κ-carrageenan-AuNPs (**b**).

**Figure 10:**
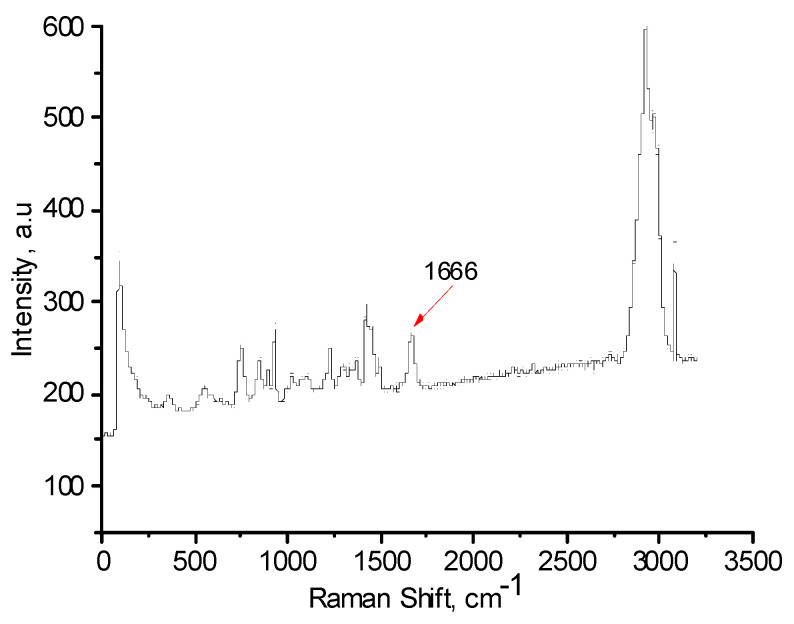
Raman spectrum of PVP 40.

**Figure 11:**
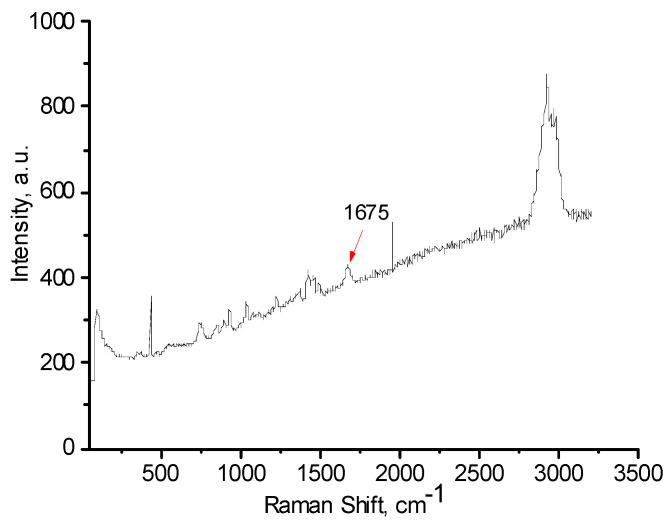
Raman spectrum of PVP 40 kDa-AuNPs.

FTIR and Raman spectra of the polymers, with and without AuNPs, were performed in order to evaluate the functional groups of polymers that are responsible for stabilization of AuNPs (Figure 6, Figure 7, Figure 8, Figure 9, Figure 10 and Figure 11). The peak at 1670 cm^−1^ that belongs to the C=O groups of pure PVP shifts to the low frequency region, appearing at 1662 cm^−1^ for PVP-AuNPs (Figure 6). This confirms the participation of the lactam ring in stabilization of AuNPs. FTIR spectra of pristine gellan and gellan-AuNPs are compared in Figure 7. The pristine gellan has a broad band centered at 3430 cm^−1^, which is attributed to the OH stretching vibration of saccharide units. The peak at 2923 cm^−1^ is related to the CH stretching vibration. The peaks at 1606 and 1404 cm^−1^ are typical for asymmetric and symmetric COO^−^ stretching modes. The intensive peak at 1033 cm^−1^ is assigned to C–O–C stretching vibrations. Seemingly, the carboxylic groups of gellan are responsible for stabilization of AuNPs, because the symmetric COO^−^ stretching mode at 1404 cm^−1^ is shifted to 1369 cm^−1^. Additionally, C–O–C groups of gellan may have an effect on the stabilization of AuNPs, as the band at 1038 cm^−1^ is shifted to 1033 cm^−1^. In the case of welan, the most sensitive functional groups in the stabilization of AuNPs are the symmetric (1413 cm^−1^) and asymmetric (1603 cm^−1^) vibration modes of carboxylic groups that are shifted to 1421 and 1578 cm^−1^, respectively (data not shown). Furthermore, the C–O–C group in welan at 1045 cm^−1^ is probably involved in the stabilization of AuNPs, because it shifts to 1037 cm^−1^. Stabilization of AuNPs by functional groups of pectin proceeds via esterified carboxylic groups at 1735 cm^−1^, which disappear in the case of pectin-AuNPs (Figure 8). For κ-carrageenan, a broad peak in the region of 3000 to 3600 cm^−1^ is due to the OH stretching vibration. The peaks found at 1643 (polymer bound water), 1236 (asymmetric stretching of O=S=O), and 1068 cm^−1^ (glycosidic bond in carrageenan) are the characteristic peaks of κ-carrageenan. The appearance of an additional peak at ν = 1382 cm^−1^, confirms the involvement of SO_2_ groups in the stabilization of AuNPs. Raman spectra of all polymers, excepting PVP 40 kDa, were not informative in evaluation of the ability to stabilize AuNPs due to high fluorescence. The frequency of C=O bonds of pristine PVP at 1666 cm^−1^ shifts to a higher frequency region, appearing at 1675 cm^−1^ for PVP-AuNPs (Figure 10 and Figure 11). This confirms the participation of carbonyl groups of PVP in the stabilization of AuNPs.

The authors [42] recorded the X-ray photoelectronic spectrum (XPS) of AuNPs stabilized by PVP 25 kDa in order to study the surface interaction between AuNPs and PVP molecules. The XPS spectrum of the AuNPs in the absence of a surface-encapsulating agent shows Au4f_5/2_ and Au4f_7/2_ peaks at a binding energy of 86.75 and 83.05 eV, respectively. In the presence of PVP, Au4f_5/2_ and Au4f_7/2_ peaks appear at 86.23 and 82.56 eV, respectively. Therefore, a decrease in binding energy at the Au4f peak (of approximately 0.52 eV in the 4f_5/2_ and 0.49 eV in the 4f_7/2)_ in the presence of PVP molecules reveals interfacial interaction between the AuNPs and PVP molecules, which confirms the reduction in the valence state from Au(3+) to Au(0) in the reaction medium. Details of the mechanism of HAuCl_4_ reduction in the presence of PVP have previously been described [43]. AuNPs have been suggested to form due to the reduction of metal ions by the macromolecular chains of PVP, due to the chemisorbed lactam ring.

### 3.4. Influence of the Concentration of Polysaccharides on the Size of AuNPs

In the case of polysaccharides, lower concentration is required to stabilize AuNPs because of their high molecular mass and specific rigid structure. For gellan, 0.5 wt % is a suitable concentration for stabilization of AuNPs (Table 3). The optimal concentration for stabilization of AuNPs in the presence of pectin, welan, and κ-carrageenan is 0.2 wt %. In spite of the smaller sizes of AuNPs at lower concentrations of polysaccharides they are unstable, and precipitate when left to stand. 

### 3.5. Influence of Temperature on the Size of AuNPs 

**Figure 12:**
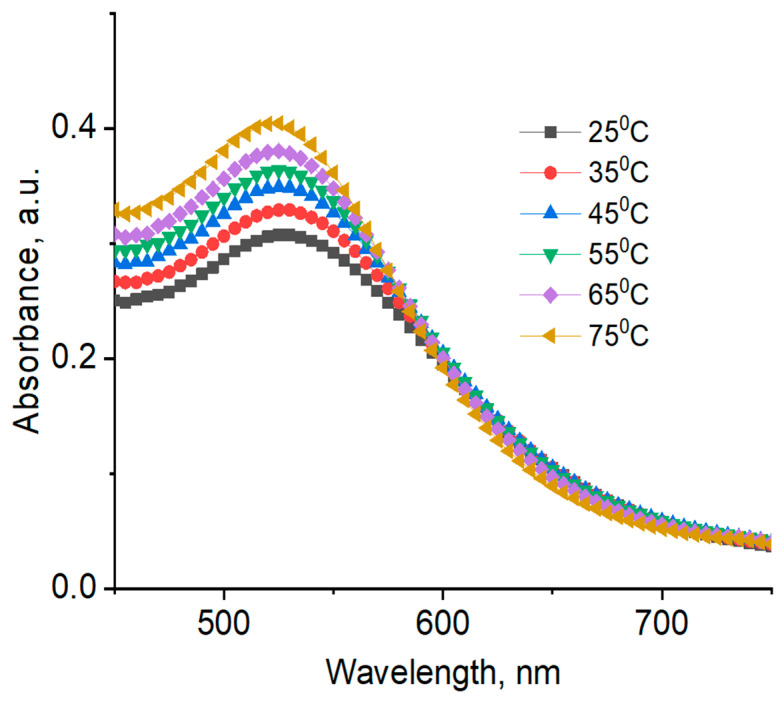
UV–Vis spectra of AuNPs synthesized and stabilized using 4 wt % PVP 40 kDa at different temperatures. [HAuCl_4_] = 0.01 mM.

**Figure 13:**
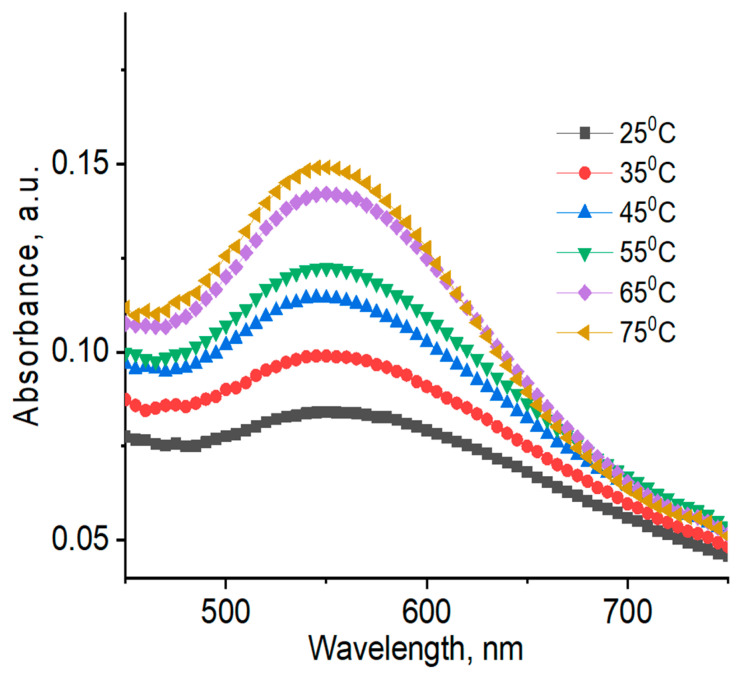
UV–Vis spectra of AuNPs synthesized and stabilized using 0.5 wt % gellan at different temperatures. [HAuCl_4_] = 0.01 mM.

UV–Vis absorption spectra and DLS data of AuNPs registered at different temperatures are shown in Figure 12; Figure 13, as well as in Table 4.

**Figure 14:**
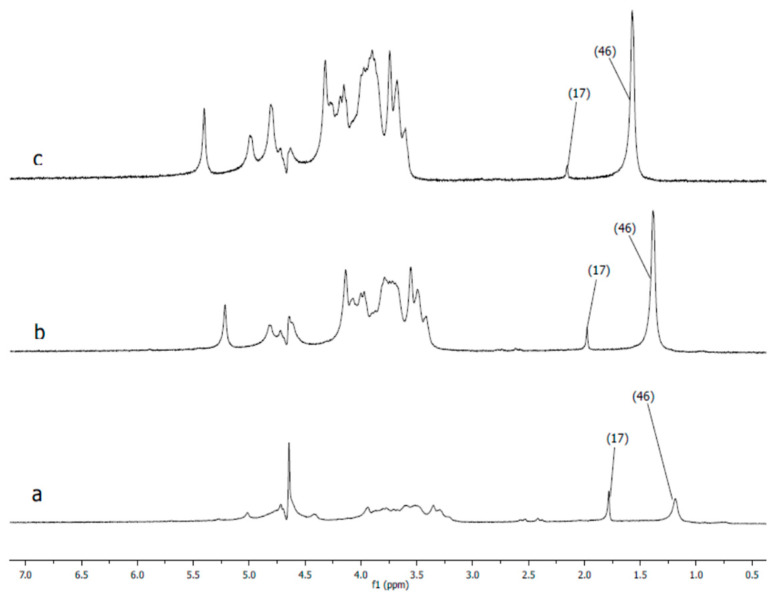
^1^H nuclear magnetic resonance (NMR) spectra of 1 wt % gellan in D_2_O at 25 (**a**), 45 (**b**), and 65 °C (**c**) [46].

As seen in Table 4, average hydrodynamic sizes of AuNPs synthesized and stabilized using natural polymers decrease as temperature is increased, which is probably due to temperature-dependent conformational changes in the polysaccharides. In order to confirm this assertion, the reduced viscosity of 0.5 wt % gellan solution was measured at temperature intervals in the range of 25 to 50 °C (data not shown) [44]. An exponential decrease in the reduced viscosity of gellan from 33 dL·g^−1^ at 25 °C, to 8 dL·g^−1^ at 50 °C was probably due to the transition of gellan from a rigid conformation at low temperatures to a disordered state at higher temperatures [45]. In addition, ^1^H NMR spectra of 1.0 wt % gellan solution in D_2_O were performed at temperature intervals in the range of 25 to 65 °C [46] (Figure 14). The proton spectra of gellan are difficult to interpret comprehensively. Therefore, the proton signals belonging to each saccharide unit were identified. Heating of 1 wt % gellan solution leads to a downfield shift of proton signals, and increases their intensity. Methyl protons of ramnopiranosol residue (H-46) were measured in solutions of 1.19, 1.39, and 1.57 ppm, at temperatures of 25, 45, and 65 °C. Methylene groups of 1,4-β-d-glucuronic acid (H-17) were recorded in solutions of 1.78, 1.98, and 2.15 ppm, at temperatures of 25, 45, and 65 °C. On the basis of these results, the authors conclude that the size of AuNPs is temperature-dependent (Table 4), based on the gradual increase of the intensity at 550 nm (Figure 13), which may be explained by the rigid to disordered conformation of gellan that affects the stabilization of AuNPs.

Average hydrodynamic sizes of AuNPs synthesized and stabilized using synthetic polymers are more or less indifferent to temperature change. Temperature-dependent SPR bands for AuNPs in PVP 40 kDa are slightly shifted to a lower wavelength region (e.g., from 525 at 25 °C, to 520 nm at 75 °C) (Figure 12).

### 3.6. Storage Stability of AuNPs 

**Figure 15:**
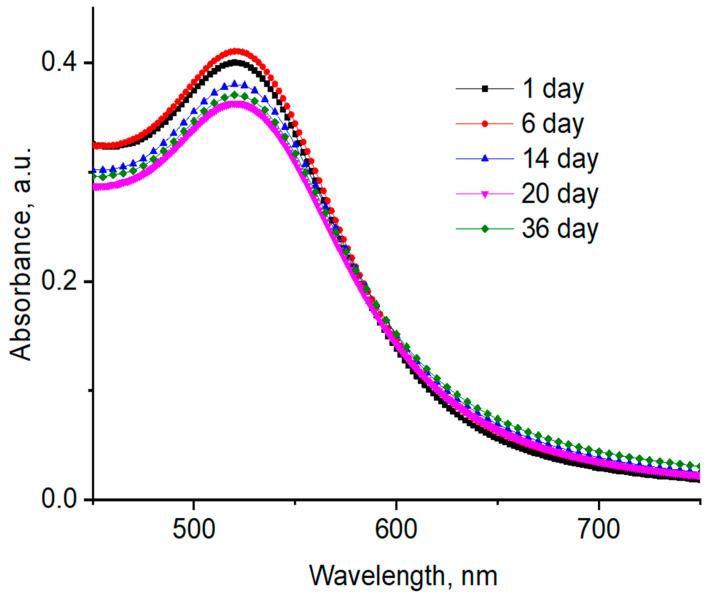
Time-dependent UV–Vis spectra of AuNPs synthesized and stabilized using PVP 40 kDa at 25 °C; [HAuCl_4_] = 0.01 mM.

The storage stability of AuNPs in the presence of PVP 40 kDa was studied after 1, 6, 14, 20, and 36 days at room temperature (Figure 15). The average hydrodynamic size distribution of AuNPs in the presence of PVP 40 kDa is presented in Table 5. The results indicate that the AuNPs synthesized and stabilized using PVP 40 kDa remained stable during the storage period. Analogous results were obtained for other polymers. 

### 3.7. Characterization of AuNRs

**Figure 16:**
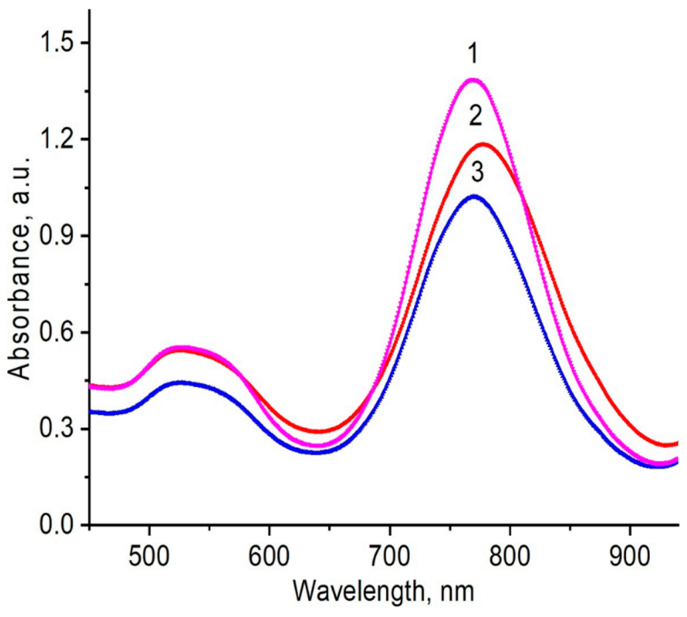
UV–Visible spectra of AuNRs synthesized and stabilized using PVP (1), PVCL (2), and gellan (3) after separation from CTAB, and washing with deionized water.

**Figure 17:**
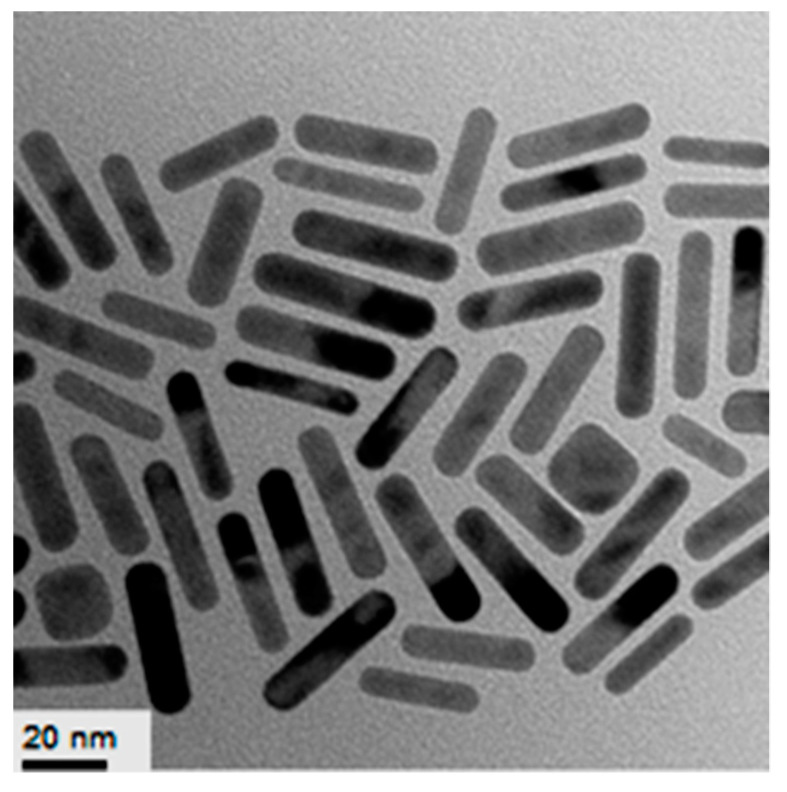
TEM images of AuNRs synthesized and stabilized using PVCL. [HAuCl_4_] = 0.5 mM.

For preparation of AuNRs the seed-mediated growth method described in the experimental section was used. Absorption spectra of polymer-coated AuNRs are placed in the NIR region at 770 nm (Figure 16). According to TEM data, the average length of the AuNRs covered by PVCL was 40–50 nm (Figure 17).

Therefore, the preliminary results show that the AuNRs may be synthesized and stabilized using both synthetic and natural polymers. The movement of the absorption spectra of AuNRs into the NIR region makes them suitable for PPTT. The selection criteria for PPTT are: (1) the ability of nanoparticles to absorb in the near-IR region; (2) the size of nanoparticles (usually less than 100 nm); (3) low toxicity (in terms of exclusion or replacement of toxic CTAB); and, (4) good biocompatibility and easy biodegradability of the polymeric coatings used for entrapment of gold nanoparticles. The results obtained are expected to be useful in matching the proper AuNP synthesis method with tumor treatments during experimentation, and in order to understand the cytotoxicity and effects of PPTT at the cellular level.

## 4. Conclusions

Polymer-protected gold nanoparticles were synthesized in the presence of various synthetic and natural water-soluble polymers using the one-pot and growth seeding approaches. Structures of functional polymers, such as lactam rings, carboxylic groups, esterified carboxylic groups, and sulfate esters act as both reducing and stabilizing agents for gold nanoparticles. The average hydrodynamic size, the intensity and position of absorption spectra, and ζ-potentials of AuNPs depend on the concentration and molecular mass of polymers, temperature, and conformation of macromolecules. Stability over a period of 36 days has been shown for AuNPs synthesized and stabilized using water-soluble polymers. The absorption spectra of polymer-coated AuNRs were shown to be in the NIR region, at 770 nm. Polymer-protected AuNPs may serve as suitable materials for biomedical applications.

## Figures and Tables

**Table 1:** Size distribution and ζ-potential of AuNPs synthesized and stabilized using synthetic and natural polymers. The standard deviations are given in parentheses.

Type of Polymers	Concentration, wt %	Range of Average Diameter, nm	ζ-Potential, mV
PVP 10 kDa	4.0	4–8 (±1)	−33.0 (±1)
PVP 40 kDa	4.0	6–9 (±1)	−28.1 (±1)
Gellan	0.5	16 (±2)	−28.5 (±1)
Pectin	0.2	11–29 (±2)	−40.3 (±1)
κ-Carrageenan	0.5	6–11 (±2)	−41.9 (±1)

**Table 2:** Influence of the concentration of PVP on the average hydrodynamic size of AuNPs. The standard deviations are given in parentheses.

C, wt %	Average Hydrodynamic Size, nm	
	PVP 10 kDa	PVP 40 kDa
0.05	-	38 (±3)
1.0	7 (±1)	10 (±1)
4.0	4 (±1)	8 (±1)

**Table 3:** Influence of the concentration of natural polymers on the average hydrodynamic size of AuNPs. The standard deviations are given in parentheses.

C, wt %	Average Hydrodynamic Size, nm			
	Gellan	Welan	Pectin	κ-Carrageenan
0.05	8 (±1)	-	9 (±2)	11 (±2)
0.2	-	18 (±2)	23 (±2)	15 (±3)
0.5	16 (±2)	30 (±3)	75 (±5)	19 (±3)
1.0	24 (±3)	42 (±4)	123 (±6)	18 (±3)

**Table 4:** Temperature-dependent average hydrodynamic size of AuNPs synthesized and stabilized using synthetic and natural polymers. The standard deviations are given in parentheses.

No.	Polymers, Concentration, wt %	Average Hydrodynamic Size of AuNPs, nm					
		Temperature, °C					
		25	35	45	55	65	75
1	Gellan, 0.5	16 (±2)	18 (±2)	11 (±2)	5 (±1)	4 (±1)	3 (±1)
2	κ-Carrageenan, 0.5	19 (±2)	19 (±2)	26 (±3)	28 (±3)	15 (±2)	11 (±2)
3	PVP 10 kDa, 4.0	2 (±1)	4 (±1)	5 (±1)	5 (±1)	5 (±1)	6 (±1)
4	PVP 40 kDa, 4.0	6 (±1)	4 (±1)	6 (±1)	5 (±1)	3 (±1)	6 (±1)

**Table 5:** The average hydrodynamic size distribution of AuNPs synthesized and stabilized using PVP 40 kDa. The standard deviations are given in parentheses.

Days	1	5	9	21	36
Diameter, nm	7–9 (±1)	7–11 (±1)	7–10 (±1)	5–26 (±2)	6–9 (±1)
